# Household water security is a mediator of household food security in a nationally representative sample of Mexico

**DOI:** 10.1017/S1368980024002684

**Published:** 2025-01-10

**Authors:** Teresa Shamah-Levy, Ignacio Méndez-Gómez-Humarán, Verónica Mundo-Rosas, Alicia Muñoz-Espinosa, Hugo Melgar-Quiñonez, Sera Lewise Young

**Affiliations:** 1 Center for Evaluation and Surveys, National Institute of Public Health of Mexico, Cuernavaca, Mexico; 2 Center For Research in Mathematics, Aguascalientes, Mexico; 3 Institute for Global Food Security, McGill University, Montreal, QC, Canada; 4 Department of Anthropology and Institute for Policy Research, Northwestern University, Evanston, IL, USA

**Keywords:** Water insecurity, Food insecurity, National survey, Mexico, Nationally representative

## Abstract

**Objective:**

Explore the relationship between water insecurity (WI) and food security and their covariates in Mexican households.

**Design:**

A cross-sectional study with nationally representative data from the National Health and Nutrition Survey-Continuous 2021 (in Spanish, ENSANUT-Continua 2021), collected data from 12 619 households.

**Setting:**

WI was measured using the Household Water Insecurity Experiences (HWISE) Scale in Spanish and adapted to the Mexican context. Food security was measured using the Latin American and Caribbean Food Security Scale. A generalised path model was used to produce two simultaneous logistical regression equations – WI (HWISE ≥ 12) and moderate-to-severe food insecurity (FI) – to understand key covariates as well as the contribution of WI to FI.

**Participants:**

The head of the household, an adult of >18 years of age, consented to participate in the survey.

**Results:**

Households experiencing WI were more likely to experience moderate-to-severe FI (OR = 2·35; 95 % CI: 2·02, 2·72). The odds of WI were lower in households with medium (OR = 0·74; 95 % CI: 0·61, 0·9) to high (OR = 0·45; 95 % CI: 0·37, 0·55) asset scores. WI also depended on the region of Mexico. FI is more prevalent in indigenous people (OR = 1·29; 95 % CI: 1·05, 1·59) and rural households (OR = 0·42; 95 % CI: 1·16, 1·73). Notably, wealth and household size did not contribute directly to FI but did so indirectly through the mediating factor of WI.

**Conclusions:**

Our study shows that there are structural factors that form part of the varied determinants of WI, which in turn is closely linked to FI.

Water security, defined as the reliable availability of adequate, acceptable and safe water, is key for basic household needs and to achieving an adequate, nutritious and high-quality diet^(1)^. Currently, the inadequate use of water globally presents significant risks to health, food and development^(2)^. Water is needed for agriculture, raising livestock and all processes of production; in 2014, nearly 70 % of the available fresh water was used to produce food^(3)^.

Even when there is enough water physically available to fulfil human needs, some vast geographical areas are near the total water scarcity, affecting millions of people, of which many are the most vulnerable, poor and disadvantaged. Therefore, the implementation and management of integrated and sustainable policies for water conservation throughout the agricultural production chain are critical.

The concept of water for food security and nutrition is gaining prominence^(4)^. Food security and nutrition includes potable water and sanitation; water used to produce, process and prepare food; and water use across all livelihood and income sectors^(5)^. The latter implies a direct pathway to economic food access, that is, food affordability. Furthermore, food security and nutrition includes the objective of sustainable management and conservation of water resources and the ecosystems that sustain them^(6)^.

In the nutrition literature, the role of water access and use in food security, nutrition and well-being has not been thoroughly documented^(7,8)^. Instead, the role of water in this literature has been focused on the role of sanitation and hygiene (WASH) in diarrheal illnesses and child development, and more recently, on environmental enteropathy^(9)^. Hydration in the context of sports nutrition has also received some attention^(8)^. Although water plays roles beyond enteric infections and homeostasis of corporal water, it has received far less attention than other essential nutrients. Water insecurity (WI) affects many other nutrition-related phenomena, such as agricultural production, food preparation and handling, dietary behaviour, dietary diversity, infant and child feeding practices and energy use^(10–14)^, and therefore deserves more attention.

It has been established that the availability of adequate and safe water is fundamental to promoting the four pillars of food security: availability, accessibility, food utilisation and stability^(15)^. For this reason, the universal guarantee of water is one of the UN Sustainable Development Goals for 2030. The corresponding 2030 Agenda states that to monitor the progress of this objective and understand the role of water in the fight to reduce food insecurity (FI), it has become critical to develop a scale to measure household WI^(16)^.

However, Young *et al.* recently documented that experiencing WI significantly increases the likelihood of also experiencing FI in several regions of the world. This suggests the importance of considering WI when designing food and nutrition policies and interventions, although more research is needed to fully understand the connections between these insecurities^(17)^.

In Mexico, experiences of household food security have been measured for the last several decades using the Latin American and Caribbean Food Security Scale (in Spanish, ELCSA)^(18,19)^. In 2012, Mexico’s National Institute of Public Health added it to the national health and nutrition survey^(20)^ and has since been measuring it regularly. In the last decade, moderate-to-severe FI in Mexico has hovered between 25·9 and 28·2 %^(20,21)^.

There is growing concern about water issues in Mexico, including scarcity, flooding and contamination^(22)^. To understand how problems with water affect public health, the National Institute of Public Health innovated by adding a national-level measurement of WI experiences in Mexico in 2021. The Household Water Insecurity Experiences (HWISE) Scale, which measures experiences of difficulties with water availability, access, use and stability^(23)^, was applied as part of the Nutrition Survey-Continuous 2021 (in Spanish, ENSANUT-Continua 2021).

The objective of this study was to evaluate the role that water security plays in food security in Mexican households. Specifically, we analysed how experiencing WI (HWISE ≥ 12) is related to moderate-to-severe FI and other covariates.

## Methods

The ENSANUT-Continua 2021 is a probabilistic and stratified national survey using cluster samples and regional representation. ENSANUT-Continua 2021 collected data from 12 619 households representing 36,476,972 Mexican households. Data were collected from August to November of 2021. The seasons of the year include summer and autumn, with the latter seeing major hurricanes in various regions of the country. Data were collected by trained enumerators in real time using tablets. Further details on the survey sample can be found elsewhere^(24)^.

Respondents generally corresponded to the person recognised as the head of household or any other household member aged 18 or older who was familiar with the household members and conditions.

### Variables

Food security was evaluated using the ELCSA, validated and adapted for Mexico^(25,26)^. It includes fifteen yes/no questions about lacking money for food, concerns about food supplies running out (mild FI), reduced diet diversity and quality (moderate FI) and limited food quantity and hunger (severe FI)^(27)^. The scale, directed at the head of the household or the member responsible for food, has a 3-month recall period. Scoring depends on positive responses and the presence of children under 18. For households without children under 18, 0 indicates food security, 1–3 mild FI, 4–6 moderate FI and 7–8 severe FI. For children under 18 years of age, 0 indicates food security, 1–5 mild FI, 6–10 moderate FI and 11–15 severe FI^(28)^.

The most recent definitions of ‘water security’ consider four dimensions: access, which refers to the ability of an individual or household to obtain water (by travelling to the water source, being able to pay for water supply, etc.). Availability considers the presence of water (‘available’). Use considers and distinguishes between the acceptability and safety of the water that individuals/households have access to (e.g. some types of water are used only for irrigation and not for human consumption). The dimension of stability or reliability simultaneously encompasses the uninterrupted existence of the three previous dimensions^(29)^. Household WI is defined as the inability to access and benefit from adequate, reliable and safe water for well-being and healthy living^(30)^. The HWISE was developed to measure the less-explored dimensions of water security. This scale is a validated tool used in several middle- and low-income countries (including some regions of Mexico) that inquired about access to and reliability of water within households.

The HWISE scale has been established as reliable, equivalent and valid in within- and cross-country analyses. Two Mexican cities were included in the validation study of HWISE^(23)^. Although the scale had already been translated into Spanish, it was considered important to pilot test the scale before including it in ENSANUT because of the cultural variety in Mexico. A group of researchers (including those who conducted the validation study) and experienced interviewers held work sessions to review and harmonise the phrases contained in each question and make the intended meaning of the items understandable. Once the first proposal of the harmonised scale was available, it was tested in 200 households in 30 states of the country, to review the comprehension of the questions and the need to include locally relevant examples. Based on the pilot study, the response to items 4, 9 and 12 was improved^(31)^.

The HWISE Scale comprises twelve questions about households’ experiences related to WI during the previous 4 weeks. The questions asked about the frequency of life-disrupting water-related problems, such as worrying about water, feeling shame about the household water situation, having to change what was eaten due to water problems and going to sleep thirsty. Possible responses are ‘never’, scored as 0; ‘rarely’, scored as 1; ‘sometimes’, scored as 2; and ‘often/always’, scored as 3. The range is 0–36; scores of 12 or higher are classified as water insecure^(32)^.

Wealth was measured using the household well-being index (HWI), which has been used in previous ENSANUT^(33)^. The HWI was constructed through principal component analysis generated using a polychoric correlation matrix^(34)^. The first component qualified as HWI, which included 40·5 and 51 % of the total variability of the included characteristics for its construction in 2012 and 2018, respectively. These were calculated using the following variables: material used to construct the dwelling (ceiling, walls and floors), number of rooms, provision of water and light services, possession of a car, number of household appliances (refrigerator, stove, washing machine, kettle, microwave oven, etc.) and the number of electronic devices (television, cable, radio and telephone). As previously described, HWI was classified into tertiles (1 = low, 2 = medium and 3 = high).

Localities with more than 2500 inhabitants were classified as urban areas, whereas those with less than 2500 were classified as rural areas.

As for the region, the ENSANUT-Continua 2021 defines nine geographic regions made up of contiguous federal entities and their population density and have been used by the Institute of Geography and Statistics to report the country’s statistics: (i) North Pacific (Baja California, Baja California Sur, Nayarit, Sinaloa and Sonora); (ii) Border (Chihuahua, Coahuila, Nuevo León and Tamaulipas); (iii) Central Pacific (Colima, Jalisco and Michoacán); (iv) Central North (Aguascalientes, Durango, Guanajuato, Querétaro, San Luís Potosí and Zacatecas); (v) Central (Hidalgo, Tlaxcala and Veracruz); (vi) Mexico City; (vii) Mexico State; (viii) South Pacific (Guerrero, Morelos, Oaxaca and Puebla); and (ix) Peninsula (Campeche, Chiapas, Quintana Roo, Tabasco and Yucatán)^(24)^.

Household size was determined based on the number of household members reported to share common household expenditures. Households in which any member spoke an indigenous language were classified as indigenous, as the previous ENSANUT.

### Statistical analysis

Variables of interest were expressed as estimated totals and proportions with 95 % CI. We described the association of experiencing WI (HWISE ≥ 12) with geographic regions, HWI and the number of household members as covariates, as well as the role of WI as a mediating factor for experiencing moderate-to-severe FI, including the contribution of determinants such as correspondence to rural areas and indigenous household head as FI covariates. A generalised path analysis model^(35)^ was used to measure the contribution of different factors to the probability of experiencing WI as a binomial response, and its contribution to moderate and severe FI was included as a binomial response, both using a logit response transformation. The estimated coefficients and their respective OR were used to support this interpretation. All analyses accounted for the design of the study in the module of complex sampling ‘svy’ and the ‘gsem’ command in STATA, v.16·1.

## Results

Of the 12 619 households visited, 12 520 had complete information of ELCSA and 12 463 on the HWISE scale. Of the population, 74·1 % had food security or mild FI, while 15·8 % had moderate FI, and 10·1 % had severe FI (Table 1). WI (HWISE scores ≥12) was experienced by 16·3 % of the population. The measure of wealth, given the use of tertiles of the HWI, suggests that the sample population is balanced across the index categories.

**Table 1.** Characteristics of sampled households in Mexico, ENSANUT-Continua 2021

Variable	*n*	Prevalence	95 % CI
HH food security			
Food secure	4712	39·2	37·8, 40·6
Mild FI	4498	34·9	33·6, 36·2
Moderate FI	2006	15·8	14·9, 16·7
Severe FI	1304	10·1	9·3, 11·0
HH water security			
Secure	10 426	83·7	81·9, 85·4
Insecure	2037	16·3	14·6, 18·2
Household well-being index^*^			
Low	4209	31·0	29·2, 32·8
Medium	4214	31·8	30·3, 33·4
High	4196	37·2	35·2, 39·2
Area of residence			
Urban	9735	79·9	78·6, 81·1
Rural	2884	20·1	18·9, 21·4
Region			
North Pacific	1589	9·9	8·9, 10·9
Border	1001	13·6	12·7, 14·7
Central Pacific	1056	10·9	10·2, 11·7
North Central	2843	12·4	11·9, 12·8
Central	951	10·2	9·6, 10·8
Mexico City	1153	7·8	7·4, 8·2
Mexico State	1199	13·0	12·5, 13·5
South Pacific	1236	12·4	11·5, 13·3
Peninsula	1591	9·9	9·4, 10·5
Indigenous background^†^			
Yes	688	5·1	4·0, 6·6
No	11 931	94·9	93·4, 96·0
Number of household members			
	12 619	3·46	1·73^†^

HH, household; FI, food insecurity.

* HWI was classified in terciles.

† Indigenous background if any member of the household spoke an indigenous language, was classified as an indigenous household.

The sample included 688 households in which the head of household spoke an indigenous language, representing 5·1 % of the national population. The average number of members per household was 3·36.

Table 2 shows conditional probabilities (expressed as percentages) of FI, given WI and other covariates. It is clear that 40·9 % of households experiencing WI showed moderate-to-severe FI and only 26·3 % were food secure. In contrast, just 22·9 % of water-secure households showed moderate-to-severe FI, while 41·9 % were food secure.

**Table 2.** Characteristics of the ENSANUT-Continua 2021 participants, by food security status

Variable	Food secure			Mild food insecurity			Moderate-to-severe food insecurity		
	*n*	%	95 % CI or sd	*n*	%	95 % CI or sd	*n*	%	95 % CI or sd
Water security									
Secure	12 617 991	41·9	40·3, 43·5	10 620 989	35·2	33·9, 36·7	6 896 685	22·9	21·5, 24·4
Insecure	1 542 139	26·3	24·0, 28·8	1 924 876	32·8	30·0, 35·8	2 394 320	40·9	37·9, 43·9
Household well-being index									
Low	2 463 543	21·8	20·3, 23·4	4 289 120	38·0	35·9, 40·1	4 543 015	40·2	38·0, 42·5
Medium	4 054 414	34·9	33·1, 36·8	4 376 296	37·7	35·8, 39·7	3 173 112	27·4	25·4, 29·4
High	7 783 822	57·3	55·1, 59·5	4 068 024	30·0	27·9, 32·1	1 725 628	12·7	11·4, 14·2
Area of residence									
Urban	12 177 673	41·8	40·1, 43·5	9 820 603	33·7	32·3, 35·1	7 142 375	24·5	23·2, 25·9
Rural	2 124 105	29·0	26·8, 31·2	2 912 837	39·7	36·7, 42·8	2 299 379	31·3	27·8, 35·1
Region									
North Pacific	1 634 074	45·5	41·8, 49·3	1 224 405	34·1	32·1, 36·1	733 698	20·4	17·3, 24·0
Border	2 633 000	53·0	47·2, 58·7	1 438 320	28·9	25·1, 33·1	898 847	18·1	14·8, 21·9
Central Pacific	1 765 438	44·2	40·7, 47·9	1 241 125	31·1	26·8, 35·8	984 038	24·7	19·6, 30·5
North Central	1 839 253	40·8	38·1, 43·6	1 685 722	37·4	35·3, 39·7	979 966	21·8	19·8, 23·8
Central	1 025 359	27·6	22·6, 33·3	1 364 283	36·7	30·5, 43·4	1 324 455	35·7	30·7, 41·0
Mexico City	1 212 414	42·7	38·5, 47·0	1 034 654	36·5	32·9, 40·2	590 299	20·8	18·2, 23·7
Mexico State	1 595 053	33·7	29·9, 37·8	1 850 419	39·1	35·7, 42·7	1 286 824	27·2	24·0, 30·7
South Pacific	1 328 451	29·5	26·3, 32·9	1 620 376	35·9	32·5, 39·5	1 561 379	34·6	30·7, 38·7
Peninsula	1 268 738	35·0	31·9, 38·2	1 274 135	35·2	32·2, 38·2	1 082 249	29·9	26·6, 33·4
Indigenous background									
Yes	516 613	27·5	23·4, 32·1	763 883	40·7	36·2, 45·4	595 474	31·7	24·3, 26·9
No	13 785 165	39·8	38·4, 41·3	11 969 557	34·6	33·3, 35·9	8 846 281	25·6	27·5, 36·4
Number of household members									
Average ± sd	14 301 778	3·17	0·04	12 733 440	3·52	0·03	9 441 755	3·42	0·04

FI was also strongly associated with low scores of HWI. The prevalence of food security was 21·8 % in households in the low-WI tertile, and up to 40·2 % reported moderate-to-severe FI. On the other hand, 57·3 % of households with high HWI scores were food secure, and only 12·7 % showed moderate-to-severe FI.

FI was also strongly associated with a low HWI score. The prevalence of food security was 21·8 % in households in the low-WI tertile, and up to 40·2 % reported moderate-to-severe FI. In contrast, 57·3 % of households with high HWI scores were food secure, and only 12·7 % showed moderate-to-severe FI.

Food security was measured at 41·8 % in urban areas and 29 % in rural areas, and the prevalence of moderate-to-severe FI was greater in rural areas (31·3 %) than in urban areas (24·5 %).

The prevalence of food security was lower in households in which the head speaks an indigenous language (27·5 %) than in their non-indigenous language-speaking counterparts (39·8 %, Table 2).

**Figure 1.**
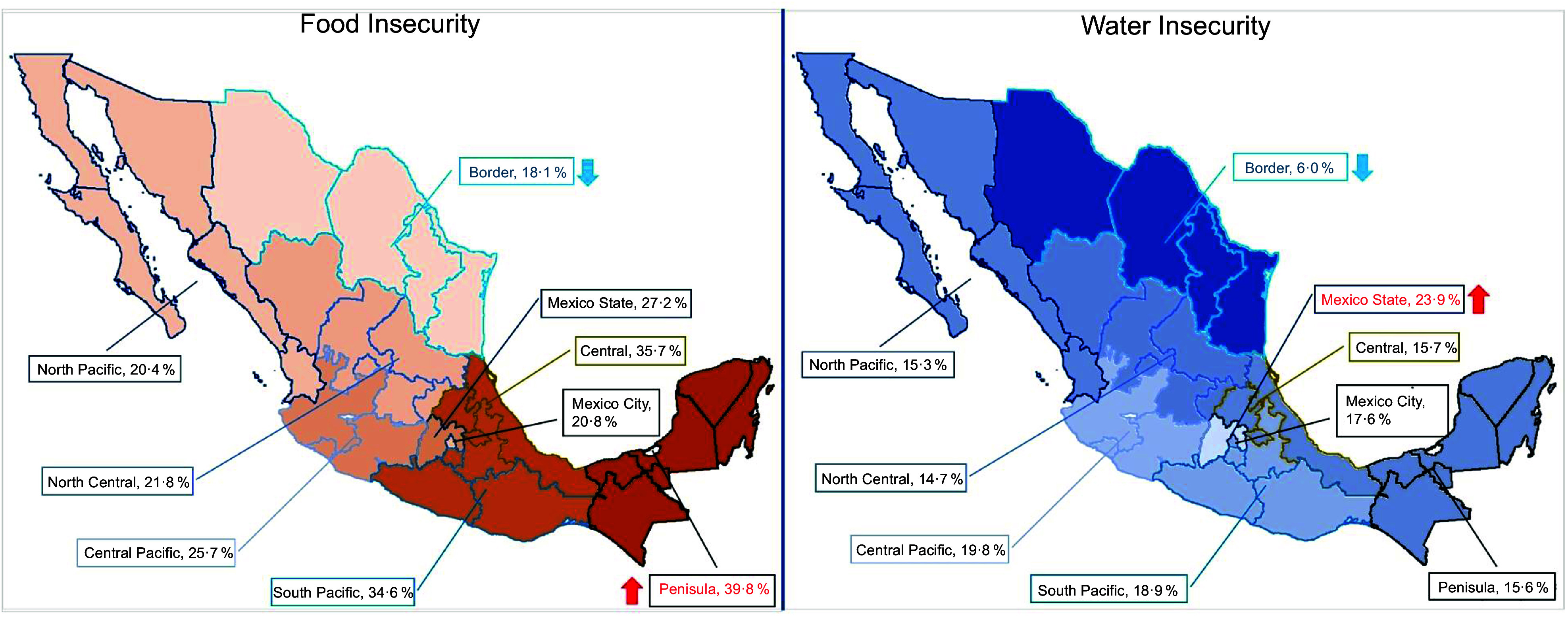
Proportion of households with moderate-to-severe food insecurity and water insecurity, by region of Mexico in the ENSANUT-Continua 2021.

By region, both FI and WI were least prevalent in the Border region (Fig. 1); this region has the highest HWI scores in the country. Even though the northern region is one of the areas with the highest economic development and the largest in terms of land area, covering over 700 000 km^2^, rivers are scarce. Nonetheless, the construction of several dams has facilitated the establishment of agricultural zones and water storage. In contrast to other regions, the indigenous groups residing in this area are few^(36)^. The Peninsula region had the highest prevalence of moderate-to-severe FI, and the Mexico State region had the highest levels of WI.

**Figure 2.**
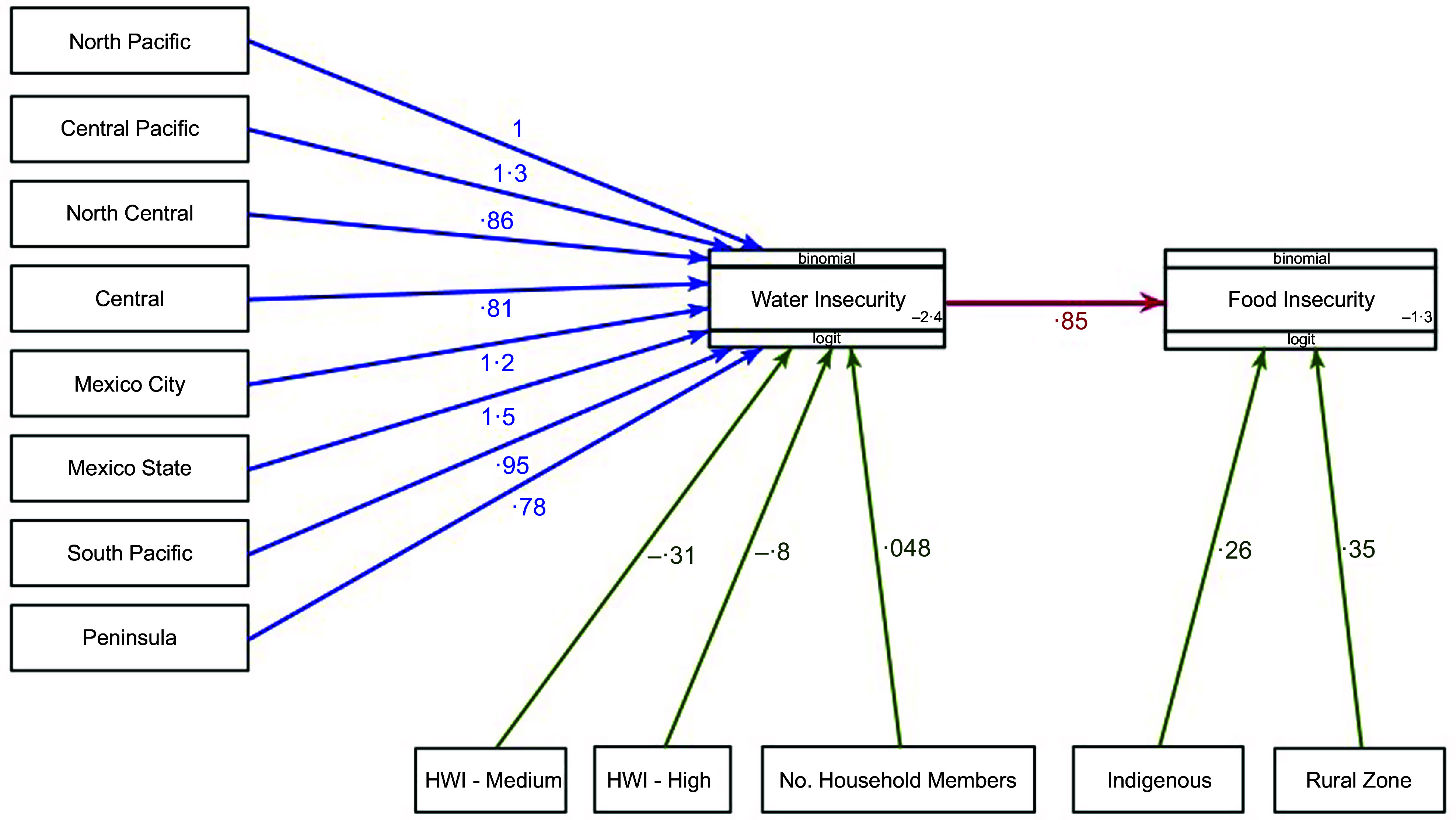
Visual representation of the general path analysis model of water and food insecurity in the ENSANUT-Continua 2021.

We utilised the information of 12 463 households with complete data of FI and WI data for generalised path analysis. The generalised path model (Fig. 2) produced two simultaneous logistical regression equations (Table 3). Equation 1 showed a significant positive association between the probability of WI and the number of household members (OR = 1·05; 95 % CI: 1·01, 1·09) and a significant positive relationship between medium and low scores of HWI and WI (OR = 1·63; 95 % CI: 1·38, 1·93 and OR = 2·22; 95 % CI: 1·82, 2·71, respectively), compared with high HWI.

**Table 3.** Generalised path model on the contributions of multiple factors to water security and food security in the ENSANUT-Continua 2021

	Coefficient	*P* > t	OR	95 % CI	
Equation 1: For water insecurity					
Number of household members	0·048	0·016	1·049	1·009	1·091
Well-being index					
Medium	0·491	0·000	1·634	1·384	1·931
Low	0·797	0·000	2·219	1·818	2·709
Region					
North Pacific	1·009	0·009	2·743	1·293	5·819
Central Pacific	1·299	0·000	3·664	1·825	7·356
North Central	0·861	0·010	2·364	1·226	4·559
Central	0·813	0·091	2·256	0·877	5·800
Mexico City	1·175	0·001	3·239	1·596	6·570
Mexico State	1·467	0·000	4·337	2·183	8·619
South Pacific	0·946	0·014	2·574	1·211	5·471
Peninsula	0·778	0·042	2·178	1·027	4·617
constant	−3·206	0·000	0·041	0·022	0·076
Equation 2: For moderate and severe food insecurity					
Water insecurity	0·854	0·000	2·348	2·024	2·723
Indigenous background	0·256	0·015	1·292	1·051	1·588
Rural area	0·347	0·001	1·415	1·155	1·734
constant	−1·304	0·000	0·272	0·250	0·295
Indirect effects of covariates on food insecurity through water insecurity as mediator					
Number of household members	0·041	0·014		0·008	0·074
Well-being index					
Medium	0·419	0·000		0·245	0·593
Low	0·680	0·000		0·472	0·889
Region					
North Pacific	0·861	0·009		0·211	1·511
Central Pacific	1·108	0·000		0·487	1·730
North Central	0·735	0·012		0·158	1·311
Central	0·694	0·112		−0·161	1·549
Mexico City	1·003	0·002		0·377	1·629
Mexico State	1·252	0·000		0·626	1·878
South Pacific	0·807	0·018		0·137	1·477
Peninsula	0·664	0·049		0·003	1·325

As we can see, WI was more prevalent in certain regions, such as the North Pacific (OR = 2·74; 95 % CI: 1·29, 5·82), Central Pacific (OR = 3·66; 95 % CI: 1·82, 7·36), Mexico State (OR = 4·33; 95 % CI: 2·18, 8·62), Mexico City (OR = 3·24; 95 % CI: 1·60, 6·57), South Pacific (OR = 2·57; 95 % CI: 1·21, 5·47), North Central (OR = 2·36; 95 % CI: 1·23, 4·56) and Peninsula (OR = 2·18; 95 % CI: 1·03, 4·62). The Border region had the lowest prevalence of WI, and only the Central region (OR = 2·26; 95 % CI: 0·88, 5·8) came close to comparing with the relatively low WI reported in the former.

Equation 2 illustrates that there is a greatly increased probability of experiencing moderate-to-severe FI for households that are WI (OR = 2·35; 95 % CI: 2·02, 2·72). The probability of experiencing moderate-to-severe FI is also greater in indigenous households (OR = 1·29; 95 % CI: 1·05, 1·59) and rural households (OR = 0·42; 95 % CI: 1·16, 1·73). Notably, wealth and household size did not contribute directly to FI but did so indirectly through the mediating factor of WI. In the bottom section of Table 3, the indirect effects of household size, HWI and region on FI through WI as a mediator are quite similar to those observed as direct effects on WI. This explains why the direct effects of this covariate on FI disappear.

## Discussion

These data demonstrate that experiences of WI have a strong positive association with moderate-to-severe FI in Mexican households. Strong associations between FI and WI have been observed in other studies, including a twenty-seven-site study in twenty-one low and middle-income countries^(23,37)^ and a twenty-five-country study conducted in collaboration with FAO^(17)^. These results are also consistent with other work that has posited WI as a plausible driver of FI^(7,38)^, including the sole study with repeated measures of FI and WI^(39)^.

Our finding that FI is more severe in rural and indigenous households aligns with previous studies in Mexico, where households in rural and indigenous communities appear to be more vulnerable^(17,40)^. With ENSANUT 2012, it was found that nationally moderate-to-severe FI affected 28·2 % of the households.

Rural or indigenous households, akin to those in the lowest HWI tertile, were particularly impacted by moderate-to-severe FI, with rates of 35·4, 42·2 and 45·2 %, respectively. Close to one-third of Mexican households experienced these more severe forms of FI, especially prevalent in rural areas of the southern states, among indigenous communities, or in conditions of poverty^(26)^. Notably, there was a decline observed in ENSANUT 2018, with rural households reporting a moderate-to-severe FI prevalence of 29·1 %^(26)^, which decreased to 27·1 %^(41)^ in 2020. However, in 2021, this figure increased to 31·3 % in rural households^(21)^.

Beyond Mexico, similar findings have been described in countries such as Guatemala and Colombia, which share similar sociodemographic characteristics and have implemented comparable strategies to address food security and WI challenges. In Guatemala, the marketing of food products has limited dietary diversity and supplanted the production and consumption of fresh nutritive foods, even in rural communities primarily dedicated to food production. This has caused the agricultural indigenous communities of Guatemala to appear much like the urban ‘food deserts’ described in higher-income countries^(42)^. In Colombia, a study among indigenous women demonstrated their vulnerability to FI and the complexities of autonomy, gender inequalities, discrimination and poverty^(43)^.

The association between WI and some of these structural factors, such as household size, area of residence and household wealth, has also been observed in previous studies^(23,44,45)^. To the best of our knowledge, differences by indigenous background have not been reported. It will be interesting to determine whether such inequalities persist elsewhere.

It will be useful to understand *how* WI shapes FI and nutrition, for example, in food production, cooking and improving the palatability and digestibility of foods or in hygiene and the prevention of food and water-borne diseases^(46)^. Evidence of this relationship so far has shown that the lack of access to water affects agricultural production, especially in rural areas where agriculture is the primary source of both income and food. Contaminated water causes illnesses such as diarrhoea and reduces the quality of food produced. Furthermore, water scarcity can limit the overall production of food and increase prices, which can further reduce the capacity for low-income households to afford food^(47)^.

Our study had certain limitations. The cross-sectional nature of the data did not allow us to infer causality. Additionally, in Mexico, no national-level indicator exists that allows the comparison of our measurements with others. Nevertheless, the data presented were derived from a representative and probabilistic national survey that previously used the ELCSA for food security measurement, and the HWISE scale used to measure WI has been previously validated in other countries, Mexico, and the context of the ENSANUT. The scale was also adapted to the country context, further strengthening the data presented that are derived from it^(31)^.

Both FI and WI are key determinants of population well-being that require immediate attention^(48,49)^. Given the close interaction between the two, it may be impossible to reduce FI without evaluating if WI is at play, which suggests that household food security interventions should include improvements in household water security^(4)^. This area requires further exploration.

It is critical to sensitise Mexican citizens and leadership to the responsible use of water, in addition to implementing strategic investments in water infrastructure and sanitation to guarantee access to safe potable water. This would not only improve the health and food security of the population but would also contribute to the national economy.

## References

[1] Jepson WE , Wutich A , Colllins SM et al. (2017) Progress in household water insecurity metrics: a cross-disciplinary approach. WIREs Water 4, e1214.

[2] Young SL , Miller JD , Frongillo EA et al. (2021) Validity of a four-item household water insecurity experiences scale for assessing water issues related to health and well-being. Am J Trop Med Hyg 104, 391–394. 33124535 10.4269/ajtmh.20-0417 PMC7790094

[3] World Bank (2017) World Bank Blogs. Chart: Globally, 70 % of Freshwater is Used for Agriculture. Available at https://blogs.worldbank.org/opendata/chart-globally-70-freshwater-used-agriculture (accessed April 2023).

[4] United Nations System Standing Committee on Nutrition (2022) Agua y Nutrición. Available at www.unscn.org/uploads/web/news/document/Water-Paper-SP-WEB.pdf (accessed July 2023).

[5] Mehta L , Oweis T , Ringler C et al. (2019) Water for Food Security, Nutrition and Social Justice. London, UK: Taylor & Francis.

[6] HLPE (2015) Water for Food Security and Nutrition. A Report by the High Level Panel of Experts on Food Security and Nutrition (HLPE). Rome: HLPE.

[7] Young SL , Frongillo EA , Jamaluddine Z et al. (2021) Perspective: the importance of water security for ensuring food security, good nutrition, and well-being. Adv Nutr 12, 1058–1073. 33601407 10.1093/advances/nmab003 PMC8321834

[8] Miller JD , Workman CL , Panchang SV et al. (2021) Water security and nutrition: current knowledge and research opportunities. Adv Nutr 12, 2525–2539. 34265039 10.1093/advances/nmab075 PMC8634318

[9] Miller JD , Vonk J , Staddon C et al. (2020) Is household water insecurity a link between water governance and well-being? A multi-site analysis. J Water Sanit Hyg Dev. 10, 320–334.

[10] Humphrey JH (2009) Child undernutrition, tropical enteropathy, toilets, and handwashing. Lancet 374, 1032–1035. 19766883 10.1016/S0140-6736(09)60950-8

[11] Popkin BM & Rosenberg IH (2011) Water, hydration and health. NIH Public Access 68, 439–458. 10.1111/j.1753-4887.2010.00304.x PMC2908954 20646222

[12] Murray B (2007) Hydration and physical performance. J Am Coll Nutr. 26, 542S–548S. 17921463 10.1080/07315724.2007.10719656

[13] Kleiner SM (1999) Water: an essential but overlooked nutrient. J Am Diet Assoc 99, 200–206. 9972188 10.1016/S0002-8223(99)00048-6

[14] Jéquier E & Constant F (2010) Water as an essential nutrient: the physiological basis of hydration. Eur J Clin Nutr 64, 115–123. 19724292 10.1038/ejcn.2009.111

[15] Nath NC (2015) Food security of Bangladesh: status, challenges and strategic policy options. Bangladesh J Polit Econ 13, 189–250.

[16] Nounkeu CD & Dharod JM (2019) Status on the scale development to measure water insecurity experiences at the household level: a narrative review. Adv Nutr 10, 864–875. 31046076 10.1093/advances/nmz008 PMC6743818

[17] Young SL , Bethancourt HJ , Frongillo EA et al. (2023) Concurrence of water and food insecurities, 25 low-and middle-income countries. Bull World Health Organ 101, 90–101. 36733622 10.2471/BLT.22.288771 PMC9874369

[18] Perez-Escamilla R , Paras P & Hromi-Fiedler A (2008) Validity of the Latin American and Caribbean household food security scale (ELCSA) in Guanajuato, Mexico. FASEB J 22, 871.

[19] Gaitán-Rossi P , Vilar-Compte M , Teruel G et al. (2021) Food insecurity measurement and prevalence estimates during the COVID-19 pandemic in a repeated cross-sectional survey in Mexico. Public Health Nutr 24, 412–421. 33050968 10.1017/S1368980020004000 PMC7653232

[20] Mundo-Rosas V , Shamah-Levy T & A Rivera-Dommarco J (2013) Epidemiología de la inseguridad alimentaria en México. Salud Publica Mex 55, S206–S213. 24626697

[21] Shamah-Levy T , Romero-Martínez M , Barrientos-Gutiérrez T et al. (2022) Encuesta Nacional de Salud y Nutrición 2021 Sobre Covid-19. Resultados Nacionales. Cuernavaca, Morelos, México: Instituto Nacional de Salud Pública.

[22] Lankao PR (2010) Water in Mexico City: what will climate change bring to its history of water-related hazards and vulnerabilities?. Environ Urban 22, 157–178.

[23] Young SL , Boateng GO , Jamaluddine Z et al. (2019) The household water insecurity experiences (HWISE) scale: development and validation of a household water insecurity measure for low-income and middle-income countries. BMJ Glob Heal 4, e001750. 10.1136/bmjgh-2019-001750 PMC6768340 31637027

[24] Romero-Martínez M , Barrientos-Gutiérrez T , Cuevas-Nasu L et al. (2021) Metodología de la encuesta nacional de salud y nutrición 2021. Salud Publica Mex 63, 813–818. 35099889 10.21149/13348

[25] Melgar-Quiñonez H , Zubieta AC , Valdez E et al. (2005) Validación de un instrumento para vigilar la inseguridad alimentaria en la Sierra de. Salud Publica Mex 47, 413–422. 16983986 10.1590/s0036-36342005000600005

[26] Mundo-Rosas V , Unar-Munguía M , Hernández-F M et al. (2019) La seguridad alimentaria en los hogares en pobreza de México: una mirada desde el acceso, la disponibilidad y el consumo. Salud Publica Mex 61, 866–875. 31869550 10.21149/10579

[27] FAO (2012) Escala Latinoamericana y Caribeña de Seguridad Alimentaria (ELCSA): Manual de uso y Aplicaciones. Roma: FAO.

[28] Melgar-Quiñonez H , Uribe MCA , Centeno ZYF et al. (2015) Características psicométricas de la escala de seguridad alimentaria ELCSA aplicada en Colombia, Guatemala y México. Segurança Aliment e Nutr 17, 48.

[29] Varis O , Keskinen M & Kummu M (2017) Four dimensions of water security with a case of the indirect role of water in global food security. Water Secur 1, 36–45.

[30] UN (2013) What is Water Insecurity?. Geneva: UN-Water.

[31] Shamah-Levy T , Mundo-Rosas V , Muñoz-Espinosa A et al. (2023) Viabilidad de una escala de experiencias de inseguridad del agua en hogares mexicanos. Salud Publica Mex 65, 219–226. 38060876 10.21149/14424

[32] Rosinger AY & Young SL (2020) The toll of household water insecurity on health and human biology: current understandings and future directions. Wiley Interdiscip Rev: Water 7, e1468.

[33] Vyas S & Kumaranayake L (2006) Constructing socio-economic status indices: how to use principal components analysis. Health Policy Plan 21, 459–468. 17030551 10.1093/heapol/czl029

[34] Drasgow F (2004) Polychoric and polyserial correlations. In Encyclopedia of Statistical Sciences, pp. 68–74 [ L Kotz , N Johnson , editors]. New York: Wiley.

[35] Tsai TL , Shau WY & Hu FC (2006) Generalized path analysis and generalized simultaneous equations model for recursive systems with responses of mixed types. Struct Equ Model 13, 229–251.

[36] Regiones de México (2002) Consejo Nacional de Educación para la Vida y el Trabajo. In México y sus Regiones Ciencias Sociales. Mexico: Secretaría de Educación Pública.

[37] Brewis A , Workman C , Wutich A et al. (2020) Household water insecurity is strongly associated with food insecurity: evidence from 27 sites in low- and middle-income countries. Am J Hum Biol 32, e23309. 31444940 10.1002/ajhb.23309 PMC9942689

[38] Rosinger AY , Bethancourt HJ , Young SL et al. (2021) The embodiment of water insecurity: injuries and chronic stress in lowland Bolivia. Soc Sci Med 291, 114490. 34662760 10.1016/j.socscimed.2021.114490 PMC8671240

[39] Boateng GO , Workman CL , Miller JD et al. (2022) The syndemic effects of food insecurity, water insecurity, and HIV on depressive symptomatology among Kenyan women. Soc Sci Med 295, 113043. 32482382 10.1016/j.socscimed.2020.113043 PMC8869838

[40] Magaña-Lemus D , Ishdorj A , Rosson CP et al. (2016) Determinants of household food insecurity in Mexico. Agric Food Econ 4, 10.

[41] Avila-Arcos MA , Humaran IMG , Morales-Ruan MDC et al. (2021) La inseguridad alimentaria y factores asociados en hogares mexicanos con casos de Covid-19. Salud Publica Mex 63, 751–762. 35099902 10.21149/13026

[42] Webb MF , Chary AN , De Vries TT et al. (2016) Exploring mechanisms of food insecurity in indigenous agricultural communities in Guatemala: a mixed methods study. BMC Nutr 2, 55.

[43] Sinclair K , Thompson-Colón T , Bastidas-Granja AM et al. (2022) Women’s autonomy and food security: connecting the dots from the perspective of Indigenous women in rural Colombia. SSM - Qual Res Heal 2, 100078.

[44] Young SL , Bethancourt HJ , Ritter ZR et al. (2022) Estimating national, demographic, and socioeconomic disparities in water insecurity experiences in low-income and middle-income countries in 2020–21: a cross-sectional, observational study using nationally representative survey data. Lancet Planet Heal 6, e880–e891. 10.1016/S2542-5196(22)00241-8 36370726

[45] Jepson WE , Stoler J , Baek J et al. (2021) Cross-sectional study to measure household water insecurity and its health outcomes in urban Mexico. BMJ Open 11, e040825. 10.1136/bmjopen-2020-040825 PMC7938997 33674365

[46] Pickering AJ & Davis J (2012) Freshwater availability and water fetching distance affect child health in sub-Saharan Africa. Environ Sci Technol 46, 9143. 10.1021/es203177v 22242546

[47] Frongillo EA (2023) Intersection of food insecurity and water insecurity. J Nutr 153, 922–923. 36848987 10.1016/j.tjnut.2023.02.024 PMC10101199

[48] Young SL , Bethancourt HJ , Cafiero C et al. (2023) Acknowledging, measuring and acting on the importance of water for food and nutrition. Nat Water 1, 825–828.

[49] Melgar-Quiñonez H , Gaitán-Rossi P , Pérez-Escamilla R et al. (2023) A declaration on the value of experiential measures of food and water insecurity to improve science and policies in Latin America and the Caribbean. Int J Equity Health 22, 184. 37670356 10.1186/s12939-023-01956-w PMC10481585

